# Effects of Biosurfactants on Enzymatic Saccharification and Fermentation of Pretreated Softwood

**DOI:** 10.3390/molecules25163559

**Published:** 2020-08-05

**Authors:** Alfredo Oliva-Taravilla, Cristhian Carrasco, Leif J. Jönsson, Carlos Martín

**Affiliations:** 1 Department of Chemistry, Umeå University, SE-901 87 Umeå, Sweden; alfredoolivat@gmail.com (A.O.-T.); leif.jonsson@umu.se (L.J.J.); 2 Instituto de Investigación y Desarrollo de Procesos Químicos (IIDEPROQ), Chemical Engineering, Faculty of Engineering, Universidad Mayor de San Andrés, La Paz 12958, Bolivia; cristhian.carrasco@gmail.com

**Keywords:** biosurfactants, cellulose, enzymatic saccharification, fermentation, quinoa saponins, steam-pretreated spruce

## Abstract

The enzymatic hydrolysis of cellulose is inhibited by non-productive adsorption of cellulases to lignin, and that is particularly problematic with lignin-rich materials such as softwood. Although conventional surfactants alleviate non-productive adsorption, using biosurfactants in softwood hydrolysis has not been reported. In this study, the effects of four biosurfactants, namely horse-chestnut escin, *Pseudomonas aeruginosa* rhamnolipid, and saponins from red and white quinoa varieties, on the enzymatic saccharification of steam-pretreated spruce were investigated. The used biosurfactants improved hydrolysis, and the best-performing one was escin, which led to cellulose conversions above 90%, decreased by around two-thirds lignin inhibition of Avicel hydrolysis, and improved hydrolysis of pretreated spruce by 24%. Red quinoa saponins (RQS) addition resulted in cellulose conversions above 80%, which was around 16% higher than without biosurfactants, and it was more effective than adding rhamnolipid or white quinoa saponins. Cellulose conversion improved with the increase in RQS addition up to 6 g/100 g biomass, but no significant changes were observed above that dosage. Although saponins are known to inhibit yeast growth, no inhibition of *Saccharomyces cerevisiae* fermentation of hydrolysates produced with RQS addition was detected. This study shows the potential of biosurfactants for enhancing the enzymatic hydrolysis of steam-pretreated softwood.

## 1. Introduction

Biochemical conversion of lignocellulosic feedstocks by process steps including pretreatment, enzymatic saccharification, and microbial fermentation is a major route to advanced biofuels and bio-based chemicals and polymers. Softwood is a major potential feedstock for biorefining processes. Softwood species include, for example, Norway spruce (*Picea abies*), which is among the predominant tree species in Nordic forestry, and different varieties of pine, which is common both in boreal forests and in plantation forestry in the Southern Hemisphere. Softwood typically contains more lignin than hardwood or agricultural residues. The high lignin content greatly contributes to making softwood recalcitrant to bioconversion [1]. Enzymatic saccharification of softwood typically results in lower yields than what is obtained with other lignocellulosic materials treated with similar enzyme dosages [2]. Although the exact mechanisms behind inhibition by lignin of enzymatic saccharification of cellulose are not yet fully elucidated [3], a major issue behind that phenomenon is non-specific, catalytically non-productive, and sometimes irreversible, binding of cellulases to lignin during enzymatic hydrolysis [4]. Lignin hydrophobicity is an important factor behind this phenomenon [5].

The non-specific adsorption of an enzyme to a substrate in a heterologous biocatalytic system, such as the enzymatic hydrolysis of cellulose, can be alleviated by introducing a surfactant into the medium [6,7]. A surfactant is an amphiphilic molecule composed of a hydrophobic portion covalently linked to a hydrophilic moiety [8]. It is known that the addition of non-ionic surfactants, i.e., those having a non-ionic hydrophilic moiety, improves the enzymatic hydrolysis of lignocellulosic materials [9]. However, most of the surfactants that have so far been used as additives for enhancing the enzymatic hydrolysis of cellulose are synthetic substances that are not biodegradable, and therefore they are toxic to the environment [7]. Using biosurfactants, which are amphiphilic substances based on renewable resources, often of microbial origin, has less impact on the environment, and it is, therefore, a more sustainable approach than using conventional synthetic surfactants [10]. Due to their specificity, biodegradability, and biocompatibility, biosurfactants have become very useful in different sectors, such as bioremediation [11], and in the pharmaceutical, cosmetic [12], food, and petroleum industries [13].

There are some examples of using biosurfactants in cellulose hydrolysis [14,15], but so far that application has been relatively poorly investigated. Two groups of biosurfactants that have attracted interest for lignocellulose bioconversion are rhamnolipids and saponins. Rhamnolipids, which contain hydrophilic rhamnose moieties linked to hydrophobic β-hydroxylated fatty-acid chains [16], have been used to prevent non-productive binding of enzymes to lignin during hydrolysis of herbaceous lignocellulosic feedstocks and hardwood [7,17,18]. Wang et al. [17] observed that the addition of rhamnolipids improved the activity and stability of cellulases, which resulted in an increase of the release of reducing sugars, and they proposed that there is more than one mechanism involved in that positive effect. Saponins, which are composed of a hydrophobic unit, a triterpenoid aglycone known as sapogenin, and at least two hydrophilic glycoside moieties [19], have recently been used to improve the enzymatic hydrolysis of residues from production of furfural from corn cobs [15,20,21].

Although the use of biosurfactants, such as rhamnolipids or saponins, has shown interesting results in the hydrolysis of herbaceous materials, their application to more recalcitrant lignocellulosic feedstocks, for instance, softwood, has, to our knowledge, not yet been reported. It is especially interesting to investigate the effects of saponins that are discarded in residual streams of agro-processing, for example, quinoa saponins. The coat covering quinoa seeds contains saponins that are related with bitterness, and, therefore, the seed coats are removed when processing quinoa grain for food use. In that operation, large amounts of saponin-rich residues are generated [22,23]. Although saponins have potential utility because of their detergent activity, toxicity against viral diseases, and cholesterol-lowering effects among other properties [24], the quinoa seed coat residue is currently underutilized. A way of giving value to that waste stream could be by extracting the saponins, and using them as biosurfactants, for instance, as enhancers of the enzymatic hydrolysis of cellulose. However, this would be a new way to utilize quinoa saponins, and because of the antimicrobial activity of saponins [25,26] including their toxic effect on yeast [27], it is not clear if it would be a viable approach.

The objective of the current work was to investigate the potential of biosurfactants for improving the enzymatic hydrolysis of cellulose contained in steam-pretreated spruce (SPS). Rhamnolipid from *Pseudomonas aeruginosa* and three sorts of saponins, namely a commercial product of high purity and two crude saponin extracts, were included in the study. Furthermore, the effects of saponin addition in the enzymatic hydrolysis step on the ethanolic fermentation by *Saccharomyces cerevisiae* were evaluated.

## 2. Results

### 2.1. Effects of Rhamnolipid and Escin on Saccharification of Avicel in the Presence of Lignin

Enzymatic saccharification of 50:50 mixtures of Avicel and lignin, with or without the addition of biosurfactants, was compared with saccharification of pure Avicel. Two commercially available biosurfactants, namely rhamnolipid from *P. aeruginosa* [16] and escin, a saponin preparation from horse-chestnut seeds [28], were investigated in experiments in which they were added at a ratio of 2 g surfactant per 100 g Avicel. The cellulose conversion during enzymatic saccharification of a mixture of Avicel and lignin corresponded to around 77.0% of the conversion achieved in the reference reaction (Avicel without lignin) (Section A of Table 1). This effect can be attributed to a large extent to the non-productive adsorption of cellulases to lignin. When biosurfactants were added, the detrimental effect of lignin decreased, and the enzymatic hydrolysis was improved, slightly (around 3%) for rhamnolipid and remarkably (more than 15%) for escin. In the presence of escin, a relative cellulose conversion of 92.4% was achieved, which corresponds to a reduction of around two thirds of the inhibition caused by lignin.

### 2.2. Effects of Rhamnolipid and Saponins on Saccharification of Steam-Pretreated Spruce

As a further investigation of the positive effects of the addition of biosurfactants observed in the enzymatic saccharification of Avicel/lignin mixtures, experiments were made with a real lignocellulosic substrate, viz. steam-pretreated spruce. The range of biosurfactants was extended with two crude saponin extracts from seed coats of red and white quinoa varieties. Crude quinoa saponins are products of potential industrial interest and they have functional similarities with escin, which was the best-performing biosurfactant in the saccharification of Avicel. The biosurfactant dosage of 2 g/100 g was used for facilitating the comparison with the Avicel experiment, but a larger dosage, 4 g/100 g, was also evaluated considering that crude saponin extracts might have a weaker effect compared to that of pure rhamnolipid and escin.

As in the experiment with Avicel/lignin mixtures (Table 1, Section A), rhamnolipid (RL) and escin improved the hydrolytic conversion of the cellulose in steam-pretreated spruce (SPS) (Table 1, Section B). The trend observed in the hydrolysis of SPS was comparable to that of Avicel hydrolysis. Escin was similarly effective at both tested dosages (improvements of 23–24%), whereas the improvement caused by RL was about twice as high (13%) at the higher dosage compared to the lower dosage (7% improvement). Both quinoa saponin extracts also improved the enzymatic saccharification, and red quinoa saponins (RQS) more than white quinoa saponins (WQS) (Table 1). As with RL, the improvements caused by the quinoa saponins were highly dose-dependent (improvements of 16% for RQS and 9% for WQS at high dosages, compared to 7% for RQS and 3% for WQS at low dosages). The highest dosage of biosurfactant addition (4 g/100 g) resulted in cellulose conversion levels of 87.4, 79.6, 81.9, and 76.8% for escin, RL, RQS, and WQS, respectively. The corresponding increases compared to the reference reaction without a biosurfactant (24, 16, and 13% for escin, RQS, and RL, respectively) were statistically significant (*p*-values ≤ 0.04).

### 2.3. Evaluation of Red Quinoa Saponins (RQS) Dosage during Enzymatic Saccharification

Although escin gave the best results, RQS, which was the second most effective among the tested biosurfactants, also deserves attention as an additive to the enzymatic hydrolysis of steam-pretreated spruce. Quinoa saponins, which can be easily extracted from quinoa seed coats, are a low-cost alternative to more expensive biosurfactants. Furthermore, using quinoa saponins in lignocellulose bioconversion might contribute to upgrading seed coats, which are an abundant and underutilized byproduct in quinoa-producing countries [29]. Therefore, the dosages of RQS under different saccharification conditions were further evaluated and optimized.

In order to elucidate how the effect of RQS changes when enzymatic saccharification is performed at enzyme and substrate loadings higher than those assayed in the first experiment, three different levels for the RQS dosage and for the loadings of enzymes and pretreated spruce were set according to a Box–Behnken factorial design. The highest RQS dosage used for the experiments with different biosurfactants (4 g/100 g biomass) was chosen as the center in the experimental design (Table 2).

**Figure 1:**
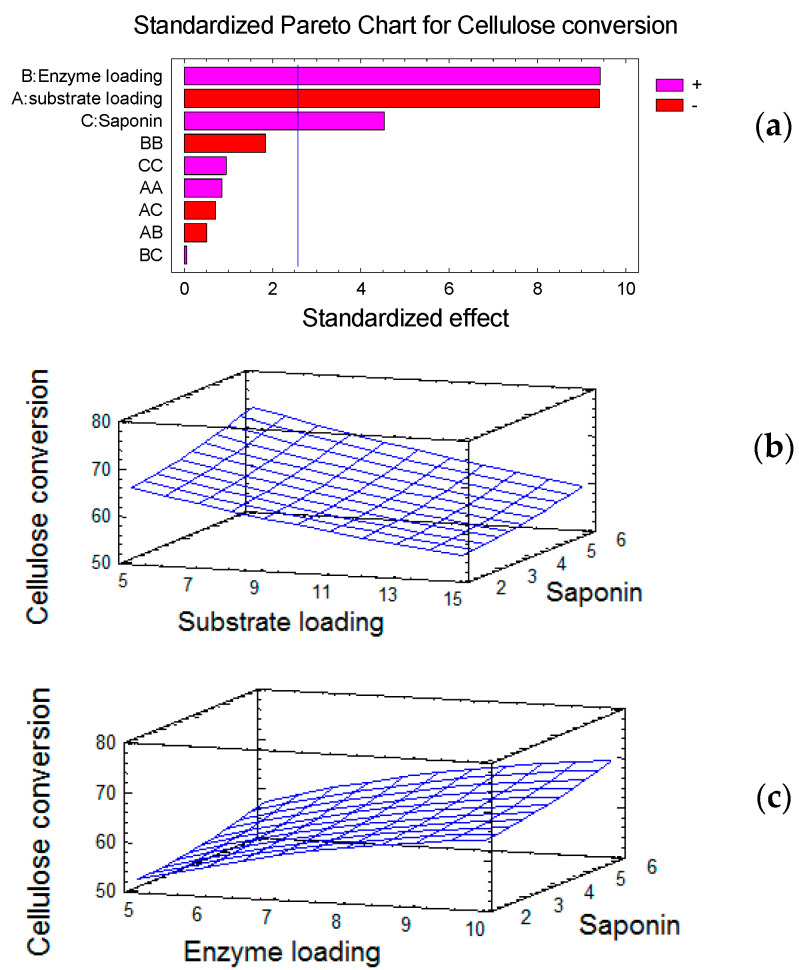
Cellulose conversion during the enzymatic hydrolysis of SPS with different RQS dosages under different loadings of enzymes and substrate. Pareto chart of standardized effects on cellulose conversion (positive, +; negative −) (**a**), and response surface plots at constant loading of enzyme (**b**) and substrate (**c**).

The Pareto chart of standardized effects shows that the dosages of both enzyme and RQS exerted directly proportional effects on cellulose conversion, whereas substrate loading affected it inversely, and none of the factor interactions exerted any important influence on the response variable (Figure 1a). The response surface plots showed that the cellulose conversion decreased with the increase in the substrate load (Figure 1b) and increased with the increase in the enzyme load (Figure 1c).

**Figure 2:**
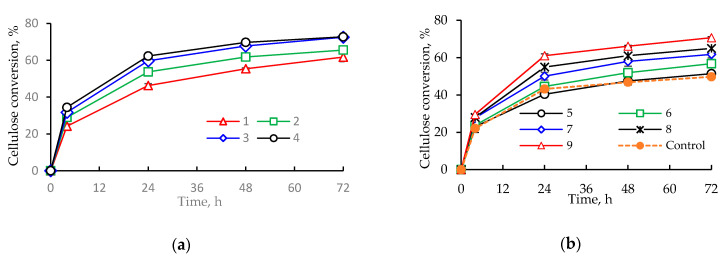

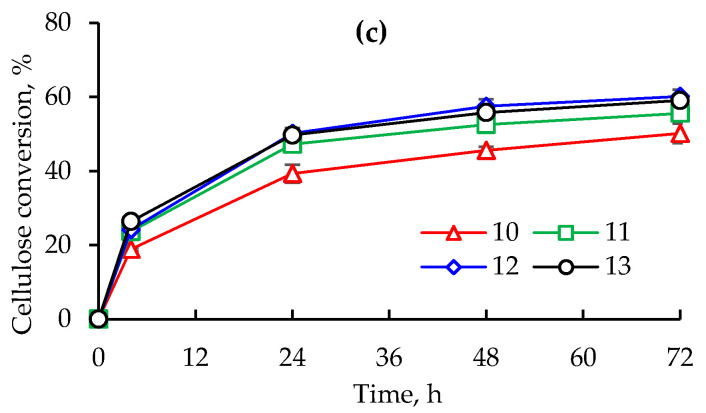
Time course of the enzymatic hydrolysis of steam-pretreated spruce at substrate loadings of 5% (**a**), 10% (**b**), and 15% (**c**). The numbers in the legend correspond to the experimental reactions as indicated in Table 2. Mean of three replicates. The error bars correspond to standard deviations.

Independently of the load of enzyme and substrate, increasing the RQS dosage always led to a clear increase in cellulose conversion. For instance, at 5% solids and 7.5 FPU/g, increasing the RQS dosage from 2 g/100 g (experimental reaction 2) to 6 g/100 g (reaction 3) enhanced the conversion by around 10% (from 66 to 73%, Figure 2a). The same applies for the experiments with 10% solids load, at either 5 or 10 FPU/g, where the conversion increased, respectively, from 51% (reaction 5) to 57% (reaction 6) and from 65% (reaction 8) to 71% (reaction 9) (Figure 2b) when the RQS dosage increased from 2 to 6 g/100 g. The effect was also evident at the highest load of substrate (15%), where increasing the saponins dosage from 2 g/100 g (reaction 11) to 6 g/100 g (reaction 12) at an enzyme loading of 7.5 FPU/g resulted in an 8% improvement (from 55.6% to 60.1%) of the cellulose conversion (Figure 2b).

Another positive effect of addition of saponins was the reduction in the enzyme dosage required for achieving similar cellulose conversion. For instance, in the experiments with 5% solids load, a 6 g/100 g RQS dosage combined with 7.5 FPU/g cellulase loading (run 3) gave a conversion comparable with that achieved by using 4 g/100 g saponins and 10 FPU/g (run 7) (Figure 2a). Similarly, at 10% solids, both 2 and 6 g/100 g RQS addition combined with the lowest cellulase loading (5 FPU/g) (reactions 5 and 6) resulted in better conversion than that achieved with a 50% higher cellulase dosage and no saponin (Control) (Figure 2b). This is in good agreement with previous reports showing that in the enzymatic hydrolysis of furfural production residues, the addition of *Gleditsia* saponin alleviated the negative effect on cellulose conversion caused by increased substrate loading [21].

Further details were revealed by examining the concentration of free protein in the reaction mixtures at the end of hydrolysis in trials with similar enzyme loadings but different saponin dosages. Independently of the amount of added enzyme, the final protein concentration was always higher for reaction mixtures with higher saponin dosages (Table 3). For instance, for 7.5 FPU/g loading and no saponin, the protein concentration at the end of the reaction was 34.6% of the initial, while in the presence of RQS, the fraction of free protein was 38.1%. Similarly, in experiments with cellulase loadings of 5 and 10 FPU/g, the fraction of protein remaining in the supernatant at the end of the saccharification reaction increased by 10% by increasing the saponin loading from 2 to 6 g/100 g.

Neither the surface response plots (Figure 1b,c) nor a complementary experiment with a wider RQS dosage range (between 2 and 12 g/100 g) revealed any optimal saponins loadings for the hydrolysis of steam-pretreated spruce. Higher dosages than those included in the experimental design only slightly affected the saccharification. For instance, the cellulose conversion achieved by an RQS addition of 8 g/100 g showed no statistical difference compared to the value obtained with 6 g/100 g, and higher dosages resulted in only marginal improvements (data not shown).

In order to estimate the fraction of RQS that adsorbed onto the lignin surface, the concentrations of RQS remaining in the medium after the saccharification reaction were assessed (Table 4). The result shows that RQS recovery after saccharification was higher when the initial dosage was low. For instance, when the initial concentration was 2 g/L, half of the RQS remained free in the reaction mixture, which points towards adsorption of the other half onto lignin. The RQS remaining in the medium after hydrolysis increased with increasing initial concentrations, indicating that after a certain dosage, no more saponin was adsorbed onto lignin.

### 2.4. Effect of Red Quinoa Saponins (RQS) on Ethanol Production by Saccharomyces cerevisiae

Although our experiments demonstrated the positive effect of biosurfactants on the enzymatic saccharification of steam-pretreated spruce, it was not clear how red quinoa saponins can affect the ethanolic fermentation of hydrolysates. Saponins can be toxic to different microorganisms. For example, *Quillaja saponaria* saponins inhibit the growth of some yeast strains [27], and saponins from quinoa seeds have been shown to exert antimicrobial, antifungal, vermicide, and anti-yeast activity [26,27]. Therefore, an evaluation of the possible inhibitory effect of RQS on the fermentation of hydrolysates was performed.

To evaluate saponin toxicity, *S. cerevisiae* Ethanol Red, a robust yeast strain especially developed for the ethanol industry [30], was used as the fermenting microorganism, and two parallel sets of experiments were run. In the first set of fermentations, aimed at evaluating the effect of the remaining saponins from that added in the enzymatic hydrolysis, spruce hydrolysates were used as the fermentation medium. In the second experimental series, focusing on the effect of the saponins added directly at the start of the fermentation, synthetic media supplemented with RQS were used. The RQS additions to the synthetic medium were equivalent to the amounts added before the enzymatic reaction for preparing the hydrolysates.

**Figure 3:**
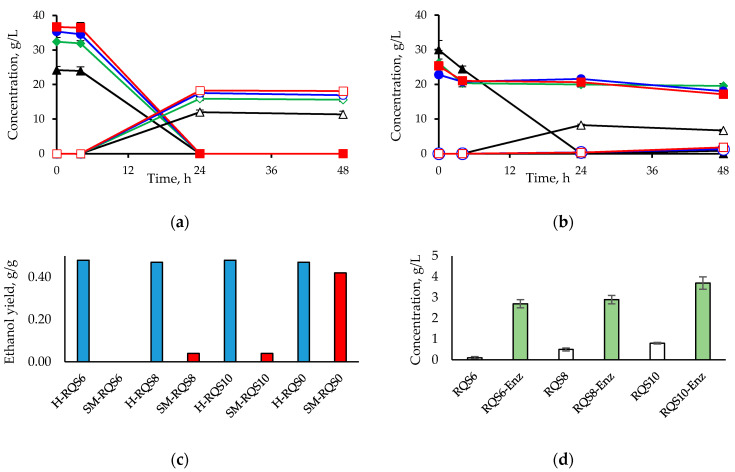
Concentration of glucose (filled symbols) and ethanol (open symbols) during fermentation of hydrolysates produced by enzymatic hydrolysis of SPS in presence of RQS (**a**) and synthetic media with directly-added RQS (**b**). RQS dosages: 6 g/100 g biomass (rhombs), 8 g/100 g biomass (circles), 10 g/100 g biomass (squares), RQS-free control (triangles). Ethanol yield in the fermentations of hydrolysates (blue bars) and synthetic media (red bars) (**c**). Final glucose concentration after incubation of RQS with (green bars) and without (white bars) cellulases (**d**). RQS concentrations in the synthetic media are equivalent to the dosages used in hydrolysis. Mean of three replicates. The error bars correspond to standard deviations.

The glucose consumption and ethanol production during the course of the fermentation of the hydrolysates showed that no inhibition occurred independently of the RQS dosage used in the hydrolysis (Figure 3a). There was a short lag phase at the beginning of the fermentation, but it was not caused by saponins since it was observed also in the RQS-free hydrolysate. After that, glucose was consumed at a high rate in all the hydrolysates, and it was depleted after 24 h. Ethanol concentrations of around 12 g/L were reached in the fermentation of the RQS-free hydrolysate, and between 16 and 18 g/L in the RQS-containing hydrolysates. That divergence was due to different initial glucose concentrations, which were 24 g/L in the hydrolysate produced without saponins supplementation, and 32–36 g/L in those produced with RQS addition (Figure 3a).

A different picture was observed when RQS was added to the synthetic medium right before yeast inoculation. A strong inhibition was noticeable in all the saponin-containing assays, whereas glucose was readily consumed in the saponin-free control (Figure 3b). In the presence of freshly added saponins, no signs of metabolic activity were detected during the first 24 h, and some minor sugar consumption and ethanol formation were evident only after 48 h. The ethanol yields per initial glucose in the synthetic medium containing freshly-added RQS were negligible compared with that of the saponin-free control, while in the hydrolysates, the yields were comparable independently of the saponin concentrations used in the hydrolysis (Figure 3c). The statistical significance of the differences between ethanol concentrations achieved in the fermentations of hydrolysates produced with RQS addition and those achieved in fermentations of synthetic media with freshly added RQS was confirmed by one-way analysis of variance (Table 5).

In an additional experiment for helping to clarify the above-stated difference, incubation of RQS with cellulases led to a dose-dependent glucose release (Figure 3d). This can be interpreted as an evidence of saponin degradation as a result of the splitting of the glycoside moieties from sapogenins during the enzymatic saccharification reaction.

## 3. Discussion

### 3.1. Effect of Biosurfactants on Enzymatic Hydrolysis of Avicel-Lignin Mixtures and Steam-Pretreated Spruce

The observed improvement in enzymatic hydrolysis can be attributed to the amphiphilic nature of the added biosurfactants, whose hydrophilic head groups would protrude into the aqueous liquid phase, while the hydrophobic tails would adsorb to exposed lignin on the surface of the pretreated solids. As a consequence, lignin–cellulase interactions are obstructed, and the protein-binding activity of lignin is reduced [15,31]. This results in an increase of the availability of effective cellulases to catalyze cellulose hydrolysis, and, therefore, the hydrolytic conversion is improved.

**Figure 4:**
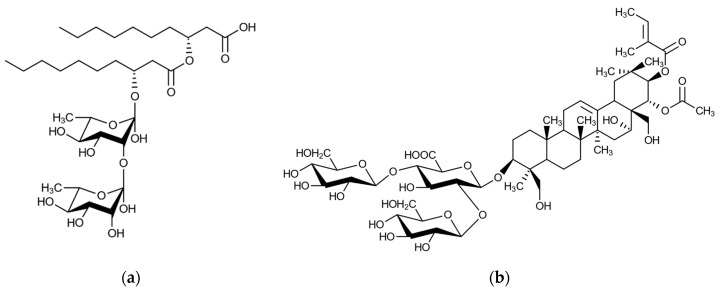
Molecular structures of rhamnolipid from *P. aeruginosa* (**a**) and horse-chestnut escin (**b**).

The differences between the effects of rhamnolipid and escin might be connected to the different chemical nature of their aglycones. Rhamnolipids are glycolipid biosurfactants with hydroxylated fatty acid tails [32], while escin, being a saponin, contains a triterpenoid sapogenin as aglycone [33]. It seems that rhamnolipid aglycone, probably due to the presence of a free carboxyl group and relatively short carbon chains compared with the more hydrophobic escin sapogenin (Figure 4), is less effective in adsorbing to lignin and thereby preventing the interference of lignin with the enzymatic saccharification of cellulose. The pH of the media used in the experiments, pH 5.0, is around or above the normal pK_a_ values of carboxylic acid groups, which might further explain why short fatty acid tails of rhamnolipids would be somewhat less effective in adsorbing to hydrophobic surfaces. The effect of *P. aeruginosa* rhamnolipid in enzymatic hydrolyses of hardwood cellulose was recently reported for eucalyptus wood chips [18], but no studies about using escin in lignocellulose hydrolysis are available to date.

The cellulose conversions achieved in the enzymatic saccharification of steam-pretreated spruce in the presence of escin, RL, and quinoa saponins (~77–87%) compare favorably with that of 74.9% reported by Xing et al. [21] for *Gleditsia* saponin-supplemented enzymatic saccharification of residues from production of furfural using corn cobs. Although the results by Xing et al. were achieved in experiments with a high solids loading (20%), our results merit attention considering that the enzyme load we used (15.4 FPU/g cellulose) was lower than theirs (30 FPU/g cellulose), our hydrolysis was shorter (72 h instead of 120 h), and the lignin content of the SPS (48.8%) was higher than that of the corn cob residues (45.3%).

Although all the tested biosurfactants exerted at least some positive effect on enzymatic hydrolysis, the magnitude of the effect was different. The differences between the effects of escin and rhamnolipid might be attributed to the different chemical functionalities of their aglycones (Figure 4). The different effect between quinoa saponins and escin saponin, which have the same chemical nature, are probably linked to their purity, i.e., the used escin was a chemically pure compound, while quinoa saponins were used in the form of crude extracts that were not subjected to any major purification. It is more difficult to explain why the effects of saponins from different quinoa varieties were so different, but one reason could be that the chemical properties of saponins differ slightly from each other.

**Figure 5:**
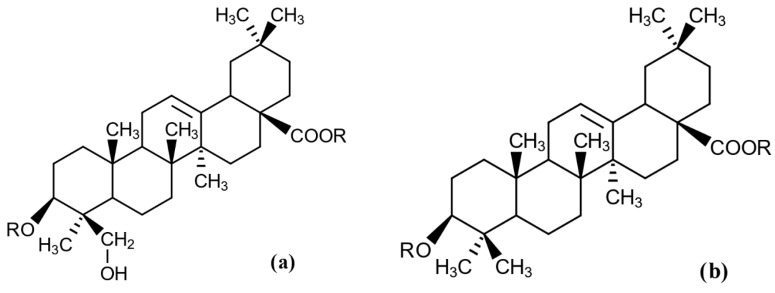
Molecular structures of sapogenins found in different varieties of quinoa: hederagenin (**a**) and oleanolic acid (**b**).

As it occurs with other biosurfactants, when saponins are added to the enzymatic hydrolysis mixture, the hydrophobic aglycone would adsorb to lignin and block unspecific adsorption of cellulases. With increasing hydrophobicity of the aglycone, the propensity of the saponin to adsorb to lignin would increase, and a further reduction in the unspecific binding of cellulases to lignin would occur. Quinoa saponins are rather heterogeneous, and they can differ in either the sapogenin moiety, the glycoside component, or both of them [24]. It can be hypothesized that saponins with highly hydrophobic aglycones would better prevent non-productive adsorption than those whose aglycones are less hydrophobic. The presence of hederagenin, a triterpenoid with certain polarity due to a free hydroxyl group (Figure 5a), as a major aglycone in WQS [34] might make them slightly less hydrophobic than those from red quinoa varieties, where sapogenins without polar functionalities, such as oleanolic acid (Figure 5b), are predominant. Furthermore, the hydrophobicity of the red quinoa pigments, which are apparently retained in the saponin extract, might also have played a role in increasing the RQS affinity for lignin. The hydrophobic binding properties of amaranthine, one of the main pigments in red quinoa, have been previously shown [35]. Amaranthine and iso-amaranthine are abundant in saponins from red quinoa but are absent in saponins from white quinoa [34].

Overall, the results confirm our hypothesis of a positive effect of biosurfactants on the enzymatic hydrolysis of pretreated softwood. As for less recalcitrant materials [14,15,17,18,20,21], RL and saponins enhanced the enzymatic hydrolysis of cellulose contained in steam-pretreated spruce. The potential of chemically synthesized surfactants for improving the enzymatic hydrolysis of pretreated softwood has been shown before [6,36], but the current study is, to the best of our knowledge, the first time that the use of biosurfactants is reported for that kind of feedstock.

### 3.2. Evaluation of Red Quinoa Saponins (RQS) Dosage during Enzymatic Saccharification

It has been shown before that the problem of catalytically non-productive binding of cellulases to lignin is more pronounced when low enzyme loadings, as those required in the industry, are used [37]. On the other hand, lignin from different biomass sources can bind cellulases to a different extent [38], and there are indications that the binding capacity of softwood lignin is higher than that of lignins from other materials [39]. Some of our experiments with pretreated softwood were conducted with cellulase loadings as low as 5 FPU/g biomass, and even at those low loadings, the positive effect of RQS was evident (Figure 1c). This, together with the low cost of quinoa saponins, provides a solid argument favoring their use as additives for the large-scale bioconversion of softwood.

The higher concentrations of free protein at the end of the saccharification reactions in experiments with higher saponin dosages (Table 3) can be linked to the observed saponin-induced reduction in the enzyme requirement for achieving comparable cellulose conversion (Figure 2). This phenomenon indicates that the surfactant effect of saponin weakens the cellulase-binding capacity of lignin. This would result in more cellulase becoming available for catalyzing the saccharification of cellulose [6].

The fact that no optimal RQS dosage was found in enzymatic hydrolysis might be related to micellar phenomena and lignin saturation resulting from high saponin concentrations in water. The experiment revealed that when the saccharification reaction is carried out using increasing RQS dosages, a threshold is reached, after which no further positive effects of the added surfactant are discernible. This might be because at a high RQS dosage the critical micelle concentration is reached, and the formed micelles hinder the diffusion of reactants and restrict the enzymatic reaction [15,40]. Since the physical and physico-chemical properties of saponin micelles depend on their biological origin [41], a further study in this area for saponins from different quinoa varieties would be of interest. It is also possible that lignin gets saturated, and no more saponins are adsorbed onto its surface independently of the amount added (Table 4). Consequently, RQS dosages above the saturation threshold would not affect the enzymatic hydrolysis of cellulose. This explains why no significant improvement of the enzymatic conversion was observed with saponin dosages above 6 g/100 g. Based on this, one can consider 6 g/100 g as an optimal RQS dosage within the experimental conditions investigated in this study. Our results are in line with a recent report showing that the glucose yield in enzymatic hydrolysis of organosolv-pretreated poplar sawdust increased proportionally with the saponin dosage only until a certain threshold, and after that it levelled off [42].

### 3.3. Effect of Red Quinoa Saponins (RQS) on Ethanol Production by Saccharomyces cerevisiae

The observed inhibition of the fermentation of synthetic medium with freshly-added RQS agrees with previous studies of the effects of saponins on the yeast microflora in winemaking [27]. That toxicity is related to the increase of yeast cell membrane permeability induced by saponins when their aglycones interact with sterols and hydrophobic tails of phospholipids, damaging the integrity of the plasma membrane and promoting the leakage of proteins and other intracellular constituents [25]. With that as background, the result that there was no inhibition of the fermentation of hydrolysates that were produced using RQS as a supplement was surprising and interesting. The different response of yeast to the saponin added to the preceding saccharification compared to that directly added to the fermentation reaction can be attributed to transformations happening during enzymatic hydrolysis.

We hypothesize that the absence of inhibition of the fermentation of the hydrolysates might be explained by either lowered concentrations of saponins in the medium or by their decreased toxicity as a result of the chemical reactions. One explanation could be that the saponin molecules, after adsorption to the lignin on the surface of pretreated solids, remain attached to it, and are removed from the liquid phase when it is separated from the solid phase after the reaction. In that case, the actual saponins concentration in the hydrolysates subjected to fermentation would be lower than the concentration added before enzymatic hydrolysis and lower than the RQS concentration in the synthetic medium. Another explanation could be that enzymes split off RQS sugar moieties from aglycones during the saccharification reaction, which decreases the saponins concentration and their detrimental effect on plasma membrane integrity. It has been shown that removal of sugars from saponins reduces their membrane-permeabilizing ability and antimicrobial activity. For instance, the heterologous expression of tomatinase, an enzyme that cleaves sugar moieties, in *S. cerevisiae* resulted in increased resistance to saponins [43]. In the current study, splitting of saponin sugar moieties was confirmed in an experiment, where incubation of RQS with cellulases for 72 h resulted in a clear increase in the glucose concentration compared with that observed when RQS was incubated without the enzyme preparation (Figure 3d). It is evident that cellulases, or perhaps other enzymes in the enzyme preparations used, can hydrolyze the β-glucosidic bonds between the D-glucopyranosyl moieties of saponin and the triterpenoid sapogenin.

Another hypothesis that could be considered is related to the potential fermentation-enhancing capacity of the released sapogenins. It could be so those sapogenins resulting from partial splitting of saponin sugar moieties during enzymatic hydrolysis, and still remaining in the hydrolysate, could be favorable for yeast in a similar way as for structurally related substances, such as certain sterols, that provide protection against ethanol toxicity during winemaking [44].

## 4. Materials and Methods

### 4.1. Pretreatment of Material

Norway spruce chips were pretreated by SEKAB E-Technology using a 30-L stainless steel reactor operating in continuous mode in the Biorefinery Demonstration Plant (Örnsköldsvik, Sweden). Unbarked wood chips were treated at 204 °C with addition of 1.2–1.3 kg SO_2_/h, corresponding to a 2% (*w*/*w*) load of SO_2_ per dry wood chips. The residence time in the reactor was 7 min, and the resulting pH was 1.5. The slurry obtained after the pretreatment was vacuum-filtered in order to separate the pretreatment liquid and the pretreated solids, which were washed with deionized water, and stored at 4 °C. The dry weight of the pretreated solids was determined by drying triplicate aliquots in a moisture analyzer (Mettler Toledo, Greifensee, Switzerland). The pretreated solids used as substrate in the enzymatic saccharification experiments contained (mass fraction, % dry weight): cellulose, 43.9; mannan, 2.2; xylan, 1.1; lignin, 48.8.

### 4.2. Enzymes and Chemicals

The enzyme preparations used were Celluclast 1.5L, with 50 FPU (filter paper units) per mL, and Novozyme 188, with 510 IU of β-glucosidase activity per mL [procured from Sigma-Aldrich (St. Louis, MO, USA) and produced by Novozymes A/S (Bagsværd, Denmark)]. Cellulase and β-glucosidase activities were determined by the filter paper method and the *p*-nitrophenyl-β-d-glucopyranoside (*p*NPG) assay, respectively [45]. Microcrystalline cellulose (Avicel PH-101, Sigma Aldrich), hereafter referred to as Avicel, was used in some enzymatic hydrolysis experiments. Rhamnolipid (RL) from *Pseudomonas aeruginosa* was acquired from Sigma-Aldrich. The saponins used in the saccharification experiments were escin from horse-chestnut (*Aesculus hippocastanum*), which is a high-purity saponin commercialized by Sigma-Aldrich, and raw saponins from red (RS) and white (WS) varieties of royal quinoa (*Chenopodium quinoa* Willd.). Raw saponins were kindly provided by Irupana Andean Organic Food S.A. (El Alto, Bolivia) and further sieved (1–1.7 mm) using a portable sieve shaker at the Instituto de Investigación y Desarrollo de Procesos Químicos, Universidad Mayor de San Andrés (La Paz, Bolivia). Saponin from *Quillaja saponaria* bark, supplied by Sigma-Aldrich, was used for analytical purposes. The used lignin was produced at Thünen Institute of Wood Research (Hamburg, Germany) by sulfuric acid-assisted organosolv pulping of beech wood [46].

### 4.3. Microorganism and Media

*S. cerevisiae* Ethanol Red (Fermentis Ltd., Marcq-en-Baroeul, France) was used as the fermentative organism. Yeast precultures in Erlenmeyer flasks (100 mL) were obtained by cultivation in 25 mL of growth medium containing (in g/L) glucose (20), yeast extract (5), and Bacto Peptone (5). The cultivation was pursued for 16 h at 150 rpm and 28 °C in an Ecotron orbital incubator (INFORS HT, Bottmingen, Switzerland). The cells were separated by centrifugation at 9000 rpm and 20 °C for 10 min, washed with sterile deionized water, and then suspended in sodium chloride 0.9% (*w/v*) right before inoculation.

### 4.4. Enzymatic Saccharification of Avicel in Presence of Lignin and Biosurfactants

Around 50 mg (DW, dry weight) Avicel and 50 mg lignin were suspended in 50 mM citrate buffer (pH 5.0) in 2-mL Eppendorf tubes giving a final solids load of 5% DW (*w*/*w*). The enzyme preparations Celluclast 1.5 L and Novozyme 188 were added at dosages corresponding to 15.4 FPU and 15.6 CBU per g cellulose. Rhamnolipid and escin were added at a ratio of 2 g per 100 g of Avicel (Table 1). A reference reaction with no lignin and no surfactants was also included. The reaction mixtures were incubated at 50 °C and 180 rpm for 72 h in the Ecotron orbital incubator, and each assay was performed in triplicate. Representative samples, withdrawn at the start and the end of the reaction, were centrifuged, and the glucose concentration in the supernatants was quantitated by HPLC, and used for calculating the enzymatic conversion of cellulose.

### 4.5. Enzymatic Saccharification of Steam-Pretreated Solids in Presence of Biosurfactants

Five hundred mg (DW) pretreated solids were mixed with 50 mM citrate buffer (pH 5.0) in 15-mL Falcon tubes giving a final solids load of 5% DW (*w*/*w*). Saccharification reactions were performed using Celluclast 1.5 L and Novozyme 188 added in the same loadings and under the same conditions as described above (Section 4.4). RL and the three different sorts of saponins (escin and crude saponins from either red or white quinoa) were added simultaneously with the enzymes at loadings of 2 and 4 g/100 g pretreated solids. A reaction without any added biosurfactants was performed as control. The enzymatic conversion of cellulose was calculated according to the following expression:

(1) $$\mathrm{EC}=\frac{Glu/1.111}{CellPS*MassPS/100}$$

where: EC, enzymatic conversion, % (*w*/*w*); *Glu*, glucose mass in the hydrolysate, g; 1.111, coefficient considering water addition to anhydroglucose units during hydrolysis; *CellPS*, cellulose content in the pretreated solids, % (*w*/*w*); *MassPS*, mass of pretreated solids sample in the enzymatic hydrolysis assay, g.

### 4.6. Assessing the Red Quinoa Saponins (RQS) Dosage on Enzymatic Hydrolysis

In a first step, the effects of the RQS dosage (2, 4, and 6 g/100 g DW pretreated solids) at different enzyme loadings (5, 7.5, and 10 FPU and CBU/g substrate) and loadings of pretreated solids (5, 10, and 15% (*w*/*w*)) on cellulose conversion were evaluated using a Box–Behnken factorial design (Table 2). Standardized Pareto charts and response surface plots were used for analyzing the effects of the independent factors on the enzymatic conversion of cellulose. An experiment without saponins, and at the conditions corresponding to the central point of the experiment, was carried out as reference. As some sugar can be released from RQS during enzymatic hydrolysis, a substrate blank, i.e., a reaction with RQS and enzyme but without substrate, was used for correcting the glucose values in the rest of the experiment. An enzyme blank was also included. The hydrolysis was run following the same protocol as described above (Section 4.4), with the exception that sampling was performed at 0, 4, 24, 48, and 72 h. The software Statgraphics Plus 5.0 for Windows (Manugistics Inc., Rockville, MD, USA) was used for designing the experiment and analyzing the results.

In a second step, a wider range of saponin dosages (0, 6, 8, 10, and 12 g/100 g pretreated solids) was evaluated in a study, where the other parameters were kept constant. The loads of solids and enzymes corresponded to the values of the central point of the above-described experiment.

### 4.7. Fermentation of Synthetic Medium and Hydrolysate in Presence of Red Quinoa Saponins (RQS)

Fermentations of either the hydrolysate containing RQS added before enzymatic hydrolysis or synthetic medium with freshly added RQS were performed. The hydrolysate was produced using substrate loading 10% (*w*/*w*), enzyme loading 7.5 FPU, and 7.5 CBU/g substrate, and RQS dosages of either 6, 8, or 10 g/100 g DW substrate. The hydrolysate was supplemented with the following nutrients (in g/L): yeast extract (5), NH_4_Cl (2), KH_2_PO_4_ (1), and MgSO_4_∙7H_2_O (0.3). The synthetic medium contained (in g/L): glucose (20), yeast extract (5), and Bacto Peptone (5). The synthetic medium was supplemented with RQS in concentrations that were equivalent to the dosages used in the preparation of the hydrolysate medium. The pH of both media was adjusted to 5.0 with 10 M NaOH. The hydrolysate was filter-sterilized using Acrodisc syringe filters (Pall, Ann Arbor, MI, USA), while the synthetic medium was sterilized by autoclaving at 110 °C for 5 min. Eight mL of hydrolysate or 20 mL of synthetic medium in Erlenmeyer flasks were inoculated with 1 g/L of yeast cells, and the fermentations were carried out at 30 °C and 150 rpm for 72 h. Samples were taken after 4, 24, 48, and 72 h of fermentation. All fermentations were performed in triplicates.

### 4.8. Analytical Methods

The composition of the pretreated solids was analyzed by using two-step treatment with sulfuric acid according to the NREL protocol [47]. The sugars were quantified by high-performance anion-exchange chromatography (HPAEC) with pulsed amperometric detection (PAD). A Dionex ICS-3000 system (Sunnyvale, CA, USA) equipped with a 3 × 30 mm guard column and a 3 × 150 mm separation column (CarboPac PA20, Dionex) was used. Acid-insoluble lignin (Klason lignin) was determined gravimetrically, whereas acid-soluble lignin (ASL) was determined spectrophotometrically at 240 nm using a UV-1800 spectrophotometer (Shimadzu, Kyoto, Japan). Glucose and ethanol concentrations from enzymatic hydrolysis and fermentations were quantified by using a high-performance liquid chromatography (HPLC) instrument equipped with a refractive index detector. An Aminex HPX-87H column (Bio-Rad Labs, Hercules, CA), operated at 55 °C with 5 mM H_2_SO_4_ as mobile-phase (0.6 mL/min), was employed for separation. Saponin concentration was determined by a modification of the afrosimetric method [48] using *Q. saponaria* bark saponins as calibration standard. The method is based on measuring the height of the foam column formed after successive shakings of a saponin suspension in 50 mM citrate buffer (pH 5.0) within two 15-min intervals. Total soluble protein was determined with the Bradford method using bovine serum albumin (BSA) as standard [49]. Non-adsorbed protein (%) in supernatants was calculated as the relation between the final (after 72 h) and initial concentrations of protein according to the following equation:

(2) $$\mathrm{Non}-\mathrm{adsorbed}\;\mathrm{protein}=\left(\frac{\mathrm{Final}\;\mathrm{protein}}{\mathrm{Initial}\;\mathrm{protein}}\right)\times 100$$

One-way analysis of variance (ANOVA) was applied for evaluating the statistical difference between the results of experiments with different biosurfactant dosages. After that, post hoc t-tests were performed to determine if the difference between specific dosages was statistically significant.

### 4.9. Formatting of Chemical Structures

The molecular editing software ChemDoodle 10.3.0 (https://www.ichemlabs.com/) was used for drawing chemical structures.

## 5. Conclusions

The results of the current study showed that adding biosurfactants to enzymatic saccharification reactions reduces non-productive binding of cellulases to lignin and effectively enhances the hydrolytic conversion not only in artificial cellulose/lignin mixtures but also in steam-pretreated softwood. Saponins, both commercial escin and crude extracts from quinoa, were highly effective as enhancers of the enzymatic hydrolysis of cellulose contained in steam-pretreated spruce. Adding red quinoa saponins to the enzymatic hydrolysis of steam-pretreated spruce did not cause any inhibitory effects on the fermentation of the resulting hydrolysates using *S. cerevisiae*. Red quinoa saponins deserve further attention as a saccharification enhancer due to their effectiveness, innocuity, low cost, and relative abundance as an agricultural by-product in quinoa-producing countries.

## Figures and Tables

**Table 1:** Cellulose conversion in enzymatic hydrolysis of Avicel/lignin mixtures (A) and steam-pretreated spruce (SPS) (B) in the presence of biosurfactants.

Substrate	Surfactant	Cellulose Conversion, % (*w*/*w*) ^1^		
		D^2^: 0	D^2^: 2	D^2^: 4
**A**				
Avicel + lignin	None	76.9 (0.4)	-	-
Avicel + lignin	Rhamnolipid	-	80.2 (3.3)	-
Avicel + lignin	Escin	-	92.4 (3.1)	-
**B**				
Pretreated solids	None	70.7 (5.2)	-	-
Pretreated solids	Rhamnolipid	-	75.7 (1.1)	79.6 (1.2)
Pretreated solids	Escin	-	87.2 (0.4)	87.4 (0.8)
Pretreated solids	RQ saponin^3^	-	75.3 (2.0)	81.9 (0.5)
Pretreated solids	WQ saponin^4^	-	73.0 (0.2)	76.8 (0.7)

^1^ The values shown in section A are percentages of the conversion in the lignin-free experiment; ^2^ biosurfactant dosage (in g/100 g substrate); ^3^ crude saponin from red quinoa; ^4^ crude saponin from white quinoa. Mean of three replicates. The standard deviations are shown in parentheses.

**Table 2:** Experimental conditions used for the evaluation of the dosage of red quinoa saponins (RQS) in the enzymatic hydrolysis of steam-pretreated spruce.

Experimental Reaction	RQS Dosage, g/100 g	Biomass Loading, % (*w*/*w*)	Enzyme Loading, FPU and CBU/g dry Weight Substrate
1	4	5	5
2	2	5	7.5
3	6	5	7.5
4	4	5	10
5	2	10	5
6	6	10	5
7	4	10	7.5
8	2	10	10
9	6	10	10
10	4	15	5
11	2	15	7.5
12	6	15	7.5
13	4	15	10
Control	0	10	7.5

**Table 3:** Total soluble protein before and after enzymatic hydrolysis of steam-pretreated spruce.

Enzyme Loading, FPU/g	Saponins Dosage, g/100 g	Protein Concentration, mg/mL		Free Protein, %
		Initial	Final	
5	2	1.24 (0.01)	0.48 (0.01)	38.7
5	6	1.24 (0.01)	0.58 (0.01)	47.7
7.5	0	1.39 (0.10)	0.48 (0.01)	34.6
7.5	4	1.39 (0.10)	0.53 (0.01)	38.1
10	2	1.69 (0.10)	0.56 (0.01)	33.3
10	6	1.69 (0.10)	0.76(0.04)	43.3

**Table 4:** Change of the concentration of red quinoa saponins (RQS) after enzymatic hydrolysis.

RQS_initial_, g/L	RQS_final_, g/L	RQS Recovery, %
2.0	1.0 (±0.1)	50.0
6.0	5.0 (±0.0)	83.3
8.0	7.5 (±0.1)	93.8
10.0	9.5 (±0.1)	95.0
12.0	11.6 (±0.1)	96.6

The enzyme loading was 7.5 FPU/g 100 g.

**Table 5:** One-way ANOVA for the ethanol concentration in the fermentation of hydrolysates produced by enzymatic hydrolysis of SPS in the presence of RQS and in synthetic media with directly added RQS.

Source of Variation	Sum of Squares	Degree of Freedom	Mean Squares	*F*-Ratio	*P*-Value	F Critical Value
Between groups	1136.787	3	378.929	587.721	1.16 × 10^−19^	3.090
Within groups	12.895	20	0.645			
Total	1149.681	23				
